# Renoprotective effects of curcumin in cats with chronic kidney disease

**DOI:** 10.1093/jvimsj/aalag138

**Published:** 2026-07-16

**Authors:** Murat İlgün, Mustafa Sinan Aktaş

**Affiliations:** Pethome Veterinary Polyclinic, Istanbul 34290, Turkey; Department of Internal Medicine, Faculty of Veterinary Medicine, Atatürk University, Erzurum 25240, Turkey

**Keywords:** apoptosis, cat, chronic kidney disease, curcumin, oxidative stress, SDMA

## Abstract

**Background:**

Chronic kidney disease (CKD) in cats is characterized by oxidative stress, inflammation, and apoptosis. Curcumin has shown renoprotective effects in experimental models, but clinical evidence in cats with CKD remains limited.

**Hypothesis/Objectives:**

Evaluate whether PO curcumin supplementation (80 mg/cat/day), administered with standard treatment, favorably modulates renal, oxidative, inflammatory, and apoptotic biomarkers in cats with International Renal Interest Society (IRIS) stage 3 CKD.

**Animals:**

Sixteen client-owned cats with IRIS stage 3 CKD.

**Methods:**

Prospective, randomized trial. Cats were allocated to medical treatment alone (MT; *n* = 8) or medical treatment plus curcumin (MT + C; *n* = 8). Curcumin was administered PO at 80 mg/cat/day in 2 divided doses for 60 days.

**Results:**

Baseline renal biomarkers did not differ between groups. The primary outcome, serum symmetric dimethylarginine (SDMA) concentration, was significantly lower in the MT + C group than in the MT group on days 30, 45, and 60 (*P* < .01). Urinary protein-to-creatinine ratio decreased over time in the MT + C group and was lower than in the MT group at later time points. Total antioxidant status increased, whereas total oxidant status decreased in the MT + C group, with a significant between-group difference in total oxidant status on day 45. Findings for nuclear factor kappa B and caspase-3 were also consistent with decreased inflammatory and apoptotic activity in curcumin-treated cats.

**Conclusions and clinical importance:**

Curcumin supplementation was associated with improvement in SDMA and favorable changes in mechanistic biomarkers in cats with IRIS stage 3 CKD.

## Introduction

Chronic kidney disease (CKD) is a progressive and irreversible condition frequently diagnosed in aging cats, characterized by glomerulosclerosis, tubular atrophy, interstitial fibrosis, and chronic inflammation.^1^^,^^2^ The International Renal Interest Society (IRIS) classifies CKD into 4 stages based on serum creatinine and symmetric dimethylarginine (SDMA) concentrations, with stage 1 representing early disease and stage 4 reflecting severe azotemia and advanced renal dysfunction.^3^ Despite advances in veterinary nephrology, current treatments remain primarily palliative, aiming to slow disease progression and alleviate clinical signs.^1^^,^^4^ However, these approaches do not directly target the molecular mechanisms underlying renal injury.^1^

The diagnosis of CKD in cats is based on clinical signs, laboratory findings, and diagnostic imaging. Persistent azotemia, inadequately concentrated urine (urine specific gravity [USG] < 1.035), and increased SDMA concentrations are key markers for early detection and staging.^5^^,^^6^ According to IRIS guidelines, proteinuria and systemic blood pressure also are used for substaging.^1^^,^^3^ Whereas serum creatinine concentration traditionally has been used to evaluate renal function, SDMA may provide an earlier indication of decreased glomerular filtration rate (GFR) in some cats.^7^ Treatment strategies include renal diets, SC fluids, antihypertensives such as amlodipine, and angiotensin receptor blockers such as telmisartan.^8^^,^^9^ Management of anemia and gastrointestinal signs further improves quality of life.^10^

Oxidative stress, inflammation, and apoptosis are key contributors to CKD pathogenesis.^11^ Reactive oxygen species (ROS) generated in diseased renal tissue cause lipid peroxidation, DNA damage, and protein oxidation, leading to activation of pro-inflammatory cytokines and transcription factors such as nuclear factor kappa B (NF-κB). These cascades contribute to tubular epithelial cell death and progressive nephron loss, as demonstrated in experimental CKD models in rodents and in humans with CKD.^12^

Curcumin, a polyphenolic compound from *Curcuma longa*, has demonstrated antioxidant, anti-inflammatory, and antiapoptotic properties in rodent models of kidney injury, including ischemia–reperfusion, diabetic nephropathy, and toxin-induced nephropathy.^13–15^ These effects involve modulation of oxidative pathways, upregulation of antioxidant enzymes including superoxide dismutase (SOD), catalase (CAT), and glutathione peroxidase (GPx); inhibition of NF-κB and cytokines such as tumor necrosis factor-alpha (TNF-α), interleukin-6 (IL-6), and interleukin-1 beta (IL-1β); as well as regulation of apoptosis via Caspase-3 and B-cell lymphoma 2 (Bcl-2).^16–18^

Despite these promising properties, curcumin’s clinical efficacy in cats with CKD remains untested. Improved bioavailability using novel formulations enhances its potential for clinical use.^19^^,^^20^ We hypothesized that daily PO administration of curcumin (80 mg/cat/day), in combination with conventional treatment, would exert renoprotective effects in cats with stage 3 CKD by modulating oxidative stress, inflammation, and apoptosis.

## Materials and methods

Twenty-four client-owned cats were enrolled: 8 clinically healthy controls and 16 cats with IRIS stage 3 chronic kidney disease (CKD). Chronic kidney disease was diagnosed by persistent azotemia, USG < 1.035, compatible clinical signs, and abdominal ultrasonography. Staging was based on serum creatinine concentration (2.9-5.0 mg/dL) after stabilization; SDMA concentration (≥26 μg/dL) supported chronic renal dysfunction but was not used for staging. Cats with prerenal azotemia, concurrent systemic disease, or recent antioxidant or anti-inflammatory supplementation were excluded.

Cats with CKD were randomized to medical treatment alone (MT; *n* = 8) or medical treatment plus curcumin (MT + C; *n* = 8). Healthy controls were sampled at baseline only for disease characterization and were not used to assess treatment efficacy. The primary endpoint was longitudinal change in serum SDMA concentration. Secondary endpoints were urine protein-to-creatinine ratio (UPC), total antioxidant status (TAS), total oxidant status (TOS), NF-κB, and caspase-3; other variables were exploratory.

Both CKD groups received IRIS-guided medical management, including renal diet, fluid therapy when indicated, telmisartan for hypertension or proteinuria, lanthanum carbonate for persistent hyperphosphatemia, dietary calcium adjustment for hypercalcemia, potassium supplementation, maropitant or mirtazapine when required, and darbepoetin with iron for anemia. The MT + C cats additionally received PO curcumin (Solgar Full Spectrum Curcumin) at 80 mg/cat/day in 2 divided doses with food for 60 days. No placebo was administered to the MT group. Laboratory personnel were blinded to group allocation throughout the study.

Blood and urine were collected from cats with CKD on day 0 before treatment and on days 15, 30, 45, and 60; controls were sampled once at baseline. Additional methodologic details, including treatment protocols, sampling procedures, and analytical characteristics of the assays are provided in Supplementary Methods S1. Hematology, biochemistry, SDMA, UPC, USG, systolic blood pressure (SBP), oxidative stress markers, and inflammatory and apoptotic biomarkers were evaluated as part of the study protocol.

Statistical analyses were performed using SPSS 25.0. Longitudinal CKD outcomes were analyzed using linear mixed effects models, with Bonferroni correction applied within outcomes. Two-sided *P* < .05 was considered significant for the primary endpoint, SDMA; secondary and exploratory outcomes were interpreted as hypothesis-generating. Additional statistical details are provided in Supplementary Methods S1.

## Results

### Clinical outcomes

No significant differences were found between the MT and MT + C groups in age, sex, neuter status, or body weight (*P* = .73, *P* = .68, *P* = .71, and *P* = .62, respectively). Among cats with CKD (*n* = 16), ages ranged from 7 to 15 years, with a mean ± SD of 11.2 ± 3.4 years. The population of cats with CKD consisted predominantly of domestic shorthair cats (69%, 11/16), followed by British shorthair (6%, 1/16), Scottish fold (6%, 1/16), Persian (6%, 1/16), Siamese (6%, 1/16), and Ankara cats (6%, 1/16). Overall, 62% were male (10/16; all neutered) and 38% were female (6/16; all spayed). The most frequent clinical signs in cats with CKD were weight loss and anorexia (94%, 15/16), polyuria and polydipsia (88%, 14/16), dehydration (75%, 12/16), and lethargy (69%, 11/16). The IRIS staging was performed based on serum creatinine concentrations at baseline, with SDMA evaluated as a supportive biomarker. One cat from the MT group died on day 44, and one from the MT + C group died on day 31; both deaths were attributed to progression of CKD rather than study procedures or interventions.

The healthy control cats (*n* = 8) were 6 to 13 years of age (9.8 ± 2.6 years). The control group included 5 males (all neutered) and 3 females (all spayed). Breeds consisted of domestic shorthair (6/8), British shorthair (1/8), and Scottish fold (1/8). Body weight was 4.6 ± 0.8 kg. All control cats were clinically healthy and had an ideal body condition score.

All CKD cats received a renal diet and lanthanum carbonate as standardized baseline treatment (MT: 8/8; MT + C: 8/8). Telmisartan was administered to all hypertensive and proteinuric cats in both groups (hypertension: MT: 8/8; MT + C: 8/8; proteinuria: MT: 8/8; MT + C: 8/8). Darbepoetin treatment was required in 6 anemic cats (MT: 3/8; MT + C: 3/8), all of which also received concurrent PO iron supplementation. Potassium supplementation was administered in 3 cats (MT: 2/8; MT + C: 1/8). Appetite stimulation with mirtazapine was used in twelve cats (MT: 6/8; MT + C: 6/8), and maropitant citrate was administered for vomiting in thirteen cats (MT: 7/8; MT + C: 6/8). Overall, the distribution of baseline concurrent treatments was comparable between groups, and no new medications were introduced during the study period.

Ultrasonographic findings in CKD cats showed a consistent pattern of chronic renal change, including increased cortical echogenicity (100%, 16/16), decreased corticomedullary definition (69%, 11/16), irregular renal margins (50%, 8/16), and decreased kidney size (13%, 2/16).

### Hematologic findings

Healthy controls were included in baseline (day 0) cross-sectional comparisons to characterize disease-associated hematologic changes at study entry, whereas longitudinal treatment-related comparisons were restricted to the MT and MT + C groups. The white blood cell (WBC) counts did not differ significantly between the MT and MT + C groups at baseline or on days 15 and 30 (*P* > .05), but were significantly higher in the MT + C group on days 45 and 60 (Table 1).

**Table 1:** Mean white blood cell count (WBC), hemoglobin (HGB), hematocrit (HCT), and red blood cell count (RBC) measurements at different time points in control, medical treatment (MT), and MT + curcumin (MT + C) groups.

**Variable**	**Time**	**Control** **$\bar{\mathrm{X}} $±SD**	**MT** **$\bar{\mathrm{X}} $±SD**	**MT + C** **$\bar{\mathrm{X}} $±SD**	***P***
**WBC (×10** ^**3**^ **/μL)**	**0**	8.51 ± 0.89^b^	11.75 ± 0.57^aAB^	11.18 ± 1.13^a^	<.05
	**15**		11.19 ± 1.58^abAB^	14.95 ± 2.03^a^	<.05
	**30**		15.10 ± 2.11^aA^	13.43 ± 0.94^a^	<.05
	**45**		10.21 ± 1.53^bB^	16.26 ± 2.20^a^	<.01
	**60**		8.69 ± 0.79^bB^	15.74 ± 2.06^a^	<.01
			*P* < .05	*P* > .05	
**HGB (g/dL)**	**0**	13.10 ± 0.75	10.02 ± 0.96	9.78 ± 1.21	>.05
	**15**		9.91 ± 1.03^b^	8.51 ± 1.08^b^	<.01
	**30**		7.9 ± 1.13^b^	9.45 ± 1.07^b^	<.01
	**45**		9.05 ± 0.94^b^	8.93 ± 0.50^b^	<.01
	**60**		9.48 ± 1.02^b^	8.20 ± 0.82^b^	<.01
			*P* > .05	*P* > .05	
**HCT (%)**	**0**	38.11 ± 2.29^a^	29.74 ± 1.99^b^	27.98 ± 2.73^b^	<.05
	**15**		29.30 ± 2.62^b^	24.40 ± 2.41^b^	<.01
	**30**		24.06 ± 2.98^b^	27.15 ± 2.72^b^	<.01
	**45**		25.27 ± 3.53^b^	26.61 ± 1.49^b^	<.01
	**60**		29.00 ± 3.20^b^	24.56 ± 2.14^b^	<.01
			*P* > .05	*P* > .05	
**RBC (×10** ^**6**^ **/μL)**	**0**	9.37 ± 0.62^a^	6.70 ± 0.74^b^	5.88 ± 0.78^b^	<.01
	**15**		7.04 ± 0.79^b^	5.58 ± 0.67^b^	<.01
	**30**		5.80 ± 0.80^b^	6.54 ± 0.74^b^	<.01
	**45**		6.05 ± 0.89^b^	6.22 ± 0.26^b^	<.01
	**60**		6.63 ± 0.98^b^	5.82 ± 0.61^b^	<.01
			*P* > .05	*P* > .05	

A, B, C: Different capital letters within the same treatment group indicate significant differences across time points. a, b, c: Different lowercase letters within the same row indicate significant between-group differences based on baseline cross-sectional comparisons or the corresponding time-point comparisons between MT and MT + C, as appropriate. Healthy controls were sampled only at day 0; therefore, comparisons involving the Control group were restricted to baseline analyses. *P*-values represent overall between-group comparisons at each time point.

Hemoglobin concentration, hematocrit, and RBC count did not differ significantly between the MT and MT + C groups at any time point (*P* > .05, Table 1). Healthy controls were included in baseline (day 0) cross-sectional comparisons to characterize anemia-related changes at study entry, whereas longitudinal treatment-related comparisons were restricted to the MT and MT + C groups.

### Biochemical findings

Serum creatinine, phosphorus, potassium, and TCO_2_ concentrations did not differ significantly between the MT and MT + C groups at any time point (*P* > .05, Table 2). Serum calcium concentrations showed no consistent between-group differences between the MT and MT + C groups throughout the study period, but overall time point-specific group effects were observed at later evaluations (Table 2). In contrast, SDMA concentrations were significantly lower in the MT + C group than in the MT group on days 30, 45, and 60 (*P* < .01). At day 60, the baseline-adjusted between-group difference in SDMA (MT vs MT + C) was 15.75 μg/dL (95% CI, 10.9-20.4), indicating a clinically meaningful treatment effect. Serum creatinine concentrations remained persistently increased in both CKD groups throughout the study, with no significant within-group or between-group differences (*P* > .05; Table 2).

**Table 2:** Mean creatinine, phosphorus (P), calcium (Ca), potassium (K), total carbon dioxide (TCO₂), and symmetric dimethylarginine (SDMA) measurements at different time points in control, medical treatment (MT), and MT + curcumin (MT + C) groups.

**Variable**	**Time**	**Control** **$\bar{\mathrm{X}} $±SD**	**MT** **$\bar{\mathrm{X}} $±SD**	**MT + C** **$\bar{\mathrm{X}} $±SD**	***P***
**Creatinine (mg/dL)**	**0**	1.00 ± 0.11	3.45 ± 0.18^a^	3.63 ± 0.24^a^	<.01
	**15**		3.70 ± 0.61^a^	3.89 ± 0.75^a^	<.01
	**30**		4.57 ± 1.82^a^	3.89 ± 0.57^a^	<.01
	**45**		3.63 ± 0.42^a^	4.23 ± 0.86^a^	<.01
	**60**		3.77 ± 0.22^a^	3.35 ± 0.38^a^	<.01
			*P* > .05	*P* > .05	
**P (mg/dL)**	**0**	4.21 ± 0.45	7.68 ± 1.01^a^	9.66 ± 1.65^a^	<.05
	**15**		7.67 ± 0.82^a^	8.55 ± 1.30^a^	<.01
	**30**		8.18 ± 1.20^a^	7.20 ± 1.04^a^	<.05
	**45**		8.42 ± 1.21^a^	7.90 ± 1.34^a^	<.05
	**60**		7.91 ± 1.06^a^	7.20 ± 1.19^a^	<.05
			*P* > .05	*P* > .05	
**Ca (mg/dL)**	**0**	10.51 ± 0.25	11.48 ± 0.41	9.85 ± 0.82^B^	>.05
	**15**		12.33 ± 2.01	12.07 ± 0.65^A^	>.05
	**30**		11.47 ± 0.50	11.91 ± 0.48^A^	>.05
	**45**		11.32 ± 0.44^ab^	12.10 ± 0.43^aA^	<.05
	**60**		11.69 ± 0.18^a^	11.81 ± 0.34^aA^	<.01
			*P* > .05	*P* < .05	
**K (mEq/L)**	**0**	3.98 ± 0.20	4.15 ± 0.35	4.03 ± 0.56	>.05
	**15**		4.18 ± 0.17	4.68 ± 0.41	>.05
	**30**		4.55 ± 0.24	4.00 ± 0.17	>.05
	**45**		4.32 ± 0.06	3.86 ± 0.18	>.05
	**60**		4.20 ± 0.22	4.05 ± 0.23	>.05
			*P* > .05	*P* > .05	
**TCO**_**2**_ **(mmol/L)**	**0**	25.71 ± 0.67	27.27 ± 2.07	21.66 ± 1.94	>.05
	**15**		26.33 ± 2.44	25.70 ± 2.11	>.05
	**30**		25.36 ± 2.31	25.53 ± 1.85	>.05
	**45**		26.52 ± 2.46	26.46 ± 1.25	>.05
	**60**		30.41 ± 2.24	27.78 ± 2.54	>.05
			*P* > .05	*P* > .05	
**SDMA (μg/dL)**	**0**	8.75 ± 0.67	38.75 ± 4.20^a^	38.50 ± 3.25^aA^	<.01
	**15**		36.00 ± 2.80^a^	33.00 ± 2.84^aAB^	<.01
	**30**		40.37 ± 5.46^a^	29.83 ± 2.23^bB^	<.01
	**45**		39.42 ± 5.01^a^	28.62 ± 2.15^bB^	<.01
	**60**		43.00 ± 4.88^a^	27.25 ± 1.72^bB^	<.01
			*P* > .05	*P* < .01	

A, B, C: Different capital letters within the same treatment group indicate significant differences across time points. a, b, c: Different lowercase letters within the same row indicate significant between-group differences based on baseline cross-sectional comparisons or the corresponding time-point comparisons between MT and MT + C, as appropriate.

Healthy controls were sampled only at day 0; therefore, comparisons involving the Control group were restricted to baseline analyses.

*P*-values represent overall between-group comparisons at each time point.

### Urinalysis findings

Within-group analysis showed a significant decrease in UPC over time in the MT + C group, beginning on day 30 and persisting through days 45 and 60 (Table 3). In addition, UPC results were significantly lower in the MT + C group than in the MT group on days 30, 45, and 60. USG remained low in both treatment groups throughout the study period, with no significant temporal changes within groups (*P* > .05; Table 3).

**Table 3:** Mean urine protein-to-creatinine ratio (UPC) and urine specific gravity (USG) measurements at different time points in control, medical treatment (MT), and MT + curcumin (MT + C) groups.

**Variable**	**Time**	**Control** **$\bar{\mathrm{X}} $±SD**	**MT** **$\bar{\mathrm{X}} $±SD**	**MT + C** **$\bar{\mathrm{X}} $±SD**	***P***
**UPC**	**0**	0.03 ± 0.01	0.53 ± 0.18^a^	0.78 ± 0.14^aA^	<.01
	**15**	-	0.57 ± 0.20^a^	0.53 ± 0.14^aB^	<.05
	**30**	-	0.64 ± 0.20^a^	0.19 ± 0.03^bC^	<.01
	**45**	-	0.51 ± 0.17^a^	0.31 ± 0.07^bC^	<.05
	**60**	-	0.39 ± 0.06^a^	0.24 ± 0.04^cC^	<.01
			*P* > .05	*P* < .01	
**USG (g/mL)**	**0**	1.047 ± 0.004	1.010 ± 0.003^b^	1.011 ± 0.005^b^	<.01
	**15**	-	1.010 ± 0.002^b^	1.012 ± 0.003^b^	<.01
	**30**	-	1.011 ± 0.002^b^	1.012 ± 0.003^b^	<.01
	**45**	-	1.011 ± 0.004^b^	1.013 ± 0.003^b^	<.01
	**60**	-	1.012 ± 0.002^b^	1.015 ± 0.003^b^	<.01
			*P* > .05	*P* > .05	

A, B, C: Different capital letters within the same treatment group indicate significant differences across time points. a, b, c: Different lowercase letters within the same row indicate significant between-group differences based on baseline cross-sectional comparisons or the corresponding time-point comparisons between MT and MT + C, as appropriate.

Healthy controls were sampled only at day 0; therefore, comparisons involving the Control group were restricted to baseline analyses.

*P*-values represent overall between-group comparisons at each time point.

### Oxidative stress findings

Total antioxidant status was lower at baseline in both treatment groups than in healthy controls, and it remained unchanged through days 15 and 30. Significant increases from baseline were observed on days 45 and 60 in the MT + C group (*P* < .01) and in the MT group (*P* < .05; Table 4).

**Table 4:** Mean total antioxidant status (TAS) and total oxidant status (TOS) measurements at different time points in control, medical treatment (MT), and MT + curcumin (MT + C) groups.

**Variable**	**Time**	**Control** **$\bar{\mathrm{X}} $±SD**	**MT** **$\bar{\mathrm{X}} $±SD**	**MT + C** **$\bar{\mathrm{X}} $±SD**	***P***
**TAS (μmol Trolox equivalent/L)**	**0**	2.14 ± 0.05	1.70 ± 0.07^bB^	1.64 ± 0.04^bB^	<.01
	**15**	-	1.83 ± 0.07^bAB^	1.75 ± 0.07^bB^	<.05
	**30**	-	1.86 ± 0.09^bAB^	1.83 ± 0.04^bB^	<.01
	**45**	-	2.17 ± 0.14^A^	2.04 ± 0.07^A^	>.05
	**60**	-	2.14 ± 0.16^A^	2.23 ± 0.08^A^	>.05
			*P* < .05	*P* < .01	
**TOS (μmol H**_**2**_ **O**_**2**_ **equivalent/L)**	**0**	3.26 ± 0.06	5.14 ± 0.19^a^	5.31 ± 0.20^aA^	<.01
	**15**	-	4.99 ± 0.21^a^	4.98 ± 0.19^aAB^	<.01
	**30**	-	4.98 ± 0.34^a^	4.60 ± 0.21^aBC^	<.01
	**45**	-	4.86 ± 0.34^a^	4.28 ± 0.23^bC^	<.01
	**60**	-	4.58 ± 0.22^a^	4.21 ± 0.20^abC^	<.01
			*P* > .05	*P* < .01	

A, B, C: Different capital letters within the same treatment group indicate significant differences across time points. a, b, c: Different lowercase letters within the same row indicate significant between-group differences based on baseline cross-sectional comparisons or the corresponding time-point comparisons between MT and MT + C, as appropriate.

Healthy controls were sampled only at day 0; therefore, comparisons involving the Control group were restricted to baseline analyses.

*P*-values represent overall between-group comparisons at each time point.

Total oxidant status was increased at all time points in both treatment groups. In the MT group, TOS did not change significantly over time (*P* > .05). Although TOS in the MT + C group was lower than in the MT group on days 45 and 60, a significant between-group difference was observed only on day 45 (*P* < .01; Table 4).

### Inflammatory and apoptotic marker findings

From day 30 onward, NF-κB concentrations in the MT group remained significantly higher than baseline (*P* < .01), whereas no significant intra-group changes were observed in the MT + C group (*P* > .05). The NF-κB concentrations were significantly lower in the MT + C group than in the MT group on days 30 and 60 (Table 5).

**Table 5:** Mean nuclear factor κ B (NF-κB) and Caspase-3 measurements at different time points in control, medical treatment (MT), and MT + curcumin (MT + C) groups.

**Variable**	**Time**	**Control** **$\bar{\mathrm{X}} $±SD**	**MT** **$\bar{\mathrm{X}} $±SD**	**MT + C** **$\bar{\mathrm{X}} $±SD**	***P***
**NF-κB (ng/mL)**	**0**	2.15 ± 0.12	3.42 ± 0.63^ab^	4.71 ± 0.65^a^	<.01
	**15**	-	3.48 ± 0.58^ab^	4.44 ± 0.80^a^	<.05
	**30**	-	4.65 ± 0.48^a^	3.45 ± 0.25^b^	<.01
	**45**	-	3.62 ± 0.30^a^	3.14 ± 0.22^ab^	<.01
	**60**	-	3.82 ± 0.22^a^	2.73 ± 0.21^b^	<.01
			*P* > .05	*P* > .05	
**Caspase-3 (ng/mL)**	**0**	1.33 ± 0.08	2.57 ± 0.30^a^	2.81 ± 0.25^aA^	<.01
	**15**	-	2.80 ± 0.36^a^	2.84 ± 0.26^aA^	<.01
	**30**	-	2.81 ± 0.32^a^	2.26 ± 0.06^aAB^	<.01
	**45**	-	2.75 ± 0.55^a^	1.96 ± 0.07^abB^	<.05
	**60**	-	2.24 ± 0.75^a^	1.66 ± 0.07^bC^	<.01
			*P* > .05	*P* < .01	

A, B, C: Different capital letters within the same treatment group indicate significant differences across time points. a, b, c: Different lowercase letters within the same row indicate significant between-group differences based on baseline cross-sectional comparisons or the corresponding time-point comparisons between MT and MT + C, as appropriate.

Healthy controls were sampled only at day 0; therefore, comparisons involving the Control group were restricted to baseline analyses.

*P*-values represent overall between-group comparisons at each time point.

Caspase-3 concentrations in the MT + C group decreased progressively over time, whereas no significant intra-group changes were observed in the MT group. A significant between-group difference was observed only on day 60, with lower Caspase-3 concentrations in the MT + C group than in the MT group (Table 5).

### Systolic blood pressure findings

Systolic blood pressure was increased at baseline in both treatment groups. It did not differ significantly between the MT and MT + C groups at most time points (*P* > .05). In the MT + C group, a significant decrease in SBP was observed at day 60 compared with baseline (*P* < .05). On day 60, SBP was significantly lower in the MT + C group than in the MT group (*P* < .05).

## Discussion

We evaluated the renoprotective effects of curcumin supplementation in cats with CKD, with a focus on its impact on renal function biomarkers, oxidative stress, inflammation, and apoptosis. The findings highlight curcumin’s therapeutic potential as adjunctive treatment in cats with CKD, indicating its ability to improve SDMA concentrations, modulate oxidative balance, suppress NF-κB-mediated inflammation, and decrease apoptotic activity through caspase-3 regulation.

Renal function biomarkers such as serum creatinine and SDMA concentrations did not differ significantly between the MT and MT + C groups at baseline, confirming that the treatment groups were comparable before intervention. Throughout the study, serum creatinine concentrations remained stable in both groups, which is consistent with expectations in stage 3 CKD where nephron loss is typically advanced. In contrast, SDMA concentrations were significantly lower in the MT + C group compared with the MT group from day 30 onward. Symmetric dimethylarginine is commonly regarded as a sensitive indicator of changes in GFR in cats.^6^ Recent comparative studies however have demonstrated that SDMA does not consistently outperform creatinine when directly correlated with measured GFR in azotemic CKD cohorts.^7^^,^^21^ These findings suggest that SDMA should be interpreted within a broader physiological context rather than as a sole indicator of GFR. Curcumin has been reported to modulate renal vascular and microcirculatory function through enhancement of nitric oxide bioavailability, improvement of endothelial function, and support of cortical microvascular perfusion.^22^^,^^23^ Nevertheless, given that both SDMA and creatinine are predominantly eliminated via GFR with minimal tubular handling, changes in renal hemodynamics would be expected to influence both biomarkers. Accordingly, the observed decrease in SDMA in the absence of a parallel change in serum creatinine concentration in the MT + C group should not be interpreted as evidence of selective glomerular or tubular effects of curcumin. Rather, this pattern likely reflects differential biomarker responsiveness over a short observation period in cats with CKD. Clarification of these relationships will require future studies incorporating direct measurements of GFR.

Phosphorus and calcium dysregulation are common complications in CKD, primarily resulting from impaired renal excretion and secondary hyperparathyroidism.^24^^,^^25^ In our study, both CKD groups exhibited persistently increased serum phosphorus concentrations throughout the observation period, consistent with reports that hyperphosphatemia often progresses despite multimodal medical management in advanced CKD.^26^ The continued increase in serum phosphorus concentrations despite lanthanum use in all cats reflects the established pathophysiology of IRIS Stage 3-4 disease. In these stages, decreased functional nephron mass, increased fibroblast growth factor 23 secretion, impaired phosphate excretion, and secondary hyperparathyroidism limit the efficacy of dietary restriction and phosphate binders.^27^ Therefore, the lack of reduction in serum phosphorus concentration in our study reflects the metabolic resistance characteristic of advanced CKD rather than differences in treatment protocols or curcumin administration.

Calcium homeostasis followed a more stable pattern. Although mild fluctuations were observed at later time points, no significant differences emerged between groups, and curcumin supplementation did not produce measurable changes in serum calcium concentrations. The relatively steady calcium profile in the MT + C group aligns with current understanding that oxidative stress, inflammation, and mineral metabolism are interdependent in CKD. Experimental studies have shown that curcumin can modulate calcium regulatory mechanisms by influencing redox balance, altering transmembrane calcium flux, and supporting intracellular homeostatic signaling pathways.^28^ These mechanisms provide a coherent biological basis for interpreting the calcium patterns observed in our study.

Urinary protein-to-creatinine ratios decreased significantly in the MT + C group from day 30 onward and remained lower through day 60, whereas UPC in the MT group remained unchanged. Persistent proteinuria is a well-recognized prognostic indicator in CKD progression.^29^^,^^30^ The downward trend in UPC observed in the MT + C group aligns with the concept that modulation of inflammatory and redox-regulated pathways can influence glomerular permeability dynamics. Experimental research has demonstrated that curcumin can interact with key molecular networks governing glomerular function, including transforming growth factor-β (TGF-β), NF-κB, and oxidative stress-responsive cascades.^17^^,^^31^ These pathways are integral to maintaining endothelial stability, extracellular matrix organization, and selective permeability across the glomerular filtration barrier. Consequently, the UPC trajectory observed in the MT + C group is consistent with mechanistic patterns described in experimental nephrology and reflects a biologically plausible response within the broader framework of curcumin-mediated glomerular modulation.

Curcumin’s effects in CKD extend beyond anti-inflammatory activity and include interactions with oxidative pathways. In our study, TAS increased and TOS decreased in the MT + C group from day 30 onward, reflecting a shift toward enhanced redox balance. Curcumin has been widely documented to influence endogenous antioxidant systems, including modulation of SOD, CAT, and GPx.^18^^,^^32^ These pathways play central roles in the neutralization of ROS, stabilization of cellular redox homeostasis, and protection of renal parenchymal structures from oxidative injury. In addition, curcumin can interact with ROS through non-enzymatic processes, such as chelating transition metals involved in Fenton chemistry, thereby decreasing the formation of highly reactive radicals.^33^ The TAS/TOS profile observed in the MT + C group aligns with these established antioxidant mechanisms and supports the concept that curcumin may contribute to more favorable oxidative conditions in CKD. Given the interconnected nature of redox, inflammatory, and apoptotic pathways in renal disease, modulation of oxidative burden also may influence downstream cellular responses.^31^

Chronic inflammation plays a central role in the progression of CKD. NF-κB, a redox-sensitive transcription factor, regulates genes involved in cytokine production, leukocyte recruitment, and fibrogenesis. In our study, NF-κB concentrations remained high in the MT group, whereas NF-κB concentrations decreased in the MT + C group in the later stages of follow-up. Curcumin has been extensively characterized as an inhibitor of NF-κB signaling through stabilization of inhibitor of kappa B alpha (IκBα) and attenuation of oxidative and inflammatory cues that facilitate NF-κB activation.^32^^,^^34^ These regulatory effects influence the transcription of key cytokines (TNF-α, IL-1β, and IL-6) and contribute to a less pro-inflammatory intrarenal environment.^31^ The NF-κB profile observed in the MT + C group is consistent with curcumin-mediated modulation of redox-inflammatory pathways and supports its potential to modulate inflammatory signaling in CKD.

Apoptosis plays a fundamental role in renal cell loss and CKD progression.^35^^,^^36^ Caspase-3, a crucial executioner caspase in the apoptotic cascade, is widely recognized as a reliable biomarker of apoptosis.^16^ In our study, caspase-3 concentrations remained increased in the MT group, whereas a marked downward shift emerged in the MT + C group beginning on day 45 and continuing through day 60. Curcumin has been broadly characterized as an agent capable of influencing apoptotic regulation through modulation of mitochondrial signaling, attenuation of cytochrome c-dependent caspase activation, and stabilization of intracellular redox conditions.^37^^,^^38^ These molecular interactions are closely tied to the balance between pro-apoptotic and anti-apoptotic signaling, including Bcl-2-associated X protein and Bcl-2 regulatory dynamics, which are central to tubular epithelial cell integrity and survival.^39^ Accordingly, the caspase-3 profile observed in the MT + C group is compatible with the mechanistic framework described for curcumin-mediated modulation of apoptotic pathways and reflects a biochemical environment supportive of decreased apoptotic drive within renal tissue.

Our study had several limitations. Sample size was relatively small, which may limit the generalizability of the results. Moreover, although multiple markers were used to evaluate renal function and systemic responses, renal histopathology was not performed, which would have provided direct evidence of tissue level changes. Future studies should consider longer follow-up periods, include histologic assessments, and explore the pharmacokinetics of curcumin in feline models to optimize dosing strategies. The healthy control group was included to facilitate baseline physiological comparisons and to contextualize disease-associated alterations in biomarker profiles. However, the limited number of control animals precludes definitive conclusions regarding population-level reference values, and treatment-related inferences were therefore primarily derived from comparisons between the MT and MT + C groups. The small sample size in each study arm, dictated by the availability of eligible stage 3 CKD cases, limits statistical power and increases the risk of both false-negative and false-positive results. Therefore, our findings should be interpreted as preliminary and hypothesis-generating.

In conclusion, PO curcumin supplementation, administered at 80 mg/cat/day in 2 divided doses for 60 consecutive days in addition to conventional medical treatment, was associated with lower SDMA concentrations and favorable changes in several mechanistic biomarkers in cats with IRIS stage 3 CKD. The observed changes in oxidative stress, inflammatory, and apoptotic markers were consistent with potential adjunctive effects of curcumin, but these secondary findings should be interpreted as hypothesis-generating. Given the small sample size, these results should be considered preliminary and warrant confirmation in larger, longer-term studies incorporating direct renal functional and histopathologic assessments.

## Supplementary Material

R1_Supplementary_Table_S1_aalag138

Supplementary_Methods_S1_aalag138

## Appendix

### Materials and methods

#### Ethical approval

Our study was conducted in accordance with the ethical principles for animal experimentation and was approved by the Atatürk University Animal Experiments Local Ethics Committee (approval no. 66; approval date: April 12, 2021; meeting no. 3). Written informed consent was obtained from the owners of all participating cats before inclusion in the study.

#### Animals

Twenty-four client-owned domestic cats were enrolled in the study, including 8 clinically healthy controls and 16 cats with IRIS stage 3 CKD. Chronic kidney disease diagnosis was based on persistent azotemia and clinical findings, and IRIS staging was performed exclusively according to serum creatinine concentrations (2.9-5.0 mg/dL for Stage 3). Concentrations of SDMA (≥26 μg/dL) were evaluated as supportive biomarkers to confirm chronic renal dysfunction but were not used for staging. Cats with prerenal azotemia were stabilized before assessment, and findings inconsistent with CKD (eg, normal SDMA with persistent isosthenuria) were excluded. Diagnostic criteria also included persistent isosthenuria (USG < 1.035) and clinical signs such as polyuria, polydipsia, weight loss, and decreased appetite. All CKD cats were clinically stable and not dehydrated at the time of staging, and IRIS classification was performed after stabilization of renal variables. Cats presented with prerenal azotemia or dehydration were initially treated using fluid therapy and re-evaluated after 72 h before inclusion. Most CKD cases were previously diagnosed within the previous 3 months and had been maintained on a commercial renal diet before study enrollment. Cats with concurrent systemic diseases (eg, hyperthyroidism, diabetes mellitus, neoplasia) or recent use of antioxidant or anti-inflammatory supplements were excluded. Before enrollment, all animals underwent comprehensive physical examination, CBC, serum biochemistry, urinalysis, and abdominal ultrasonography.

#### Study design and group allocation

The primary outcome of the study was the longitudinal change in serum SDMA concentrations between the MT and MT + C groups, selected because of its clinical relevance as a sensitive marker of renal function in cats with CKD. Secondary outcomes included UPC, oxidative stress markers (TAS, TOS), and inflammatory and apoptotic biomarkers (NF-κB, Caspase-3), which were assessed to provide mechanistic context for the primary endpoint.

Twenty-four cats were assigned into 3 equal groups (*n* = 8 per group) as follows:

Control Group (Control): Clinically healthy cats without evidence of CKD, as confirmed by normal physical examination findings, normal hematologic and biochemical test results, USG > 1.035, and normal renal ultrasonographic appearance. The control group was included to facilitate baseline physiological comparisons with the CKD groups and to contextualize disease-associated alterations in biomarker profiles.

Medical treatment group (MT): Cats diagnosed with IRIS stage 3 CKD received medical treatment only, including a renal prescription diet, fluid therapy, phosphate binders, and antihypertensive agents, without any additional supplementation.

Curcumin Group (MT + C): Cats with stage 3 CKD received the same medical treatment as the MT group, and PO curcumin supplementation (Solgar® Full Spectrum Curcumin, Istanbul, Turkey) at a dosage of 80 mg/cat/day (2 divided doses) for 60 consecutive days.^40^ This formulation, designed for humans, contained micronized curcumin in liquid form (40 mg curcumin per capsule) and was administered with food to enhance absorption.

Cats were treated and monitored at a Pethome Veterinary Polyclinic. Randomization was conducted using a computer-generated sequence. No placebo was administered to the MT group, but all investigators and laboratory personnel were blinded to group allocation throughout the study.

The healthy control group was included to permit baseline cross-sectional comparisons with the CKD groups and to characterize disease-related alterations in oxidative, inflammatory, and apoptotic biomarkers at study entry, especially because standardized reference ranges for several of these biomarkers are not well established in cats. However, the healthy control group was not intended to serve as a comparator for treatment efficacy. Therefore, evaluation of therapeutic effects was based primarily on longitudinal comparisons between the MT and MT + C groups.

#### Treatment protocols

Throughout the study, cats in the medical treatment group (MT, *n* = 8) diagnosed with stage 3 CKD received comprehensive medical management in accordance with IRIS guidelines. All treatments were performed at a Pethome Veterinary Polyclinic. All cats were fed a renal prescription diet (Unique Renal® Diet, France) beginning on day 0. In dehydrated cats, Ringer’s lactate solution was administered IV based on body weight, followed by SC fluid administration every other day for maintenance. Metabolic acidosis was determined by measuring total carbon dioxide (TCO_2_) concentrations using an automated chemistry analyzer (Fuji NX 500 I, Fujifilm Corporation, Tokyo, Japan), and cats with TCO₂ concentrations < 15 mmol/L were considered acidotic.^1^ SBP was measured on days 0, 15, 30, 45, and 60 using a high-definition oscillometric device (Hasvet 838 PM, Hasvet Medical, Istanbul, Turkey). Measurements were obtained with the cat in sternal recumbency, using an appropriately sized cuff (40% of limb circumference) placed on the median portion of the forelimb. All measurements were performed in a quiet room after a 5-min acclimation period to minimize stress-induced variability. For each time point, 5 consecutive readings were recorded, and the mean value of the last 3 measurements was used for analysis. Cats with SBP ≥ 160 mmHg received telmisartan (Semintra® 4 mg/mL, Boehringer Ingelheim Vetmedica, Ingelheim, Germany) at a dosage of 2 mg/kg/day to decrease systolic pressure below 160 mmHg.^41^^,^^42^ Serum creatinine concentration and UPC ratios were evaluated on days 0, 15, 30, 45, and 60. Serum creatinine concentrations were measured using a biochemistry analyzer (Fuji NX 500 I, Fujifilm Corporation, Tokyo, Japan) and UPC ratios were determined using an automated chemistry analyzer (Catalyst One, IDEXX Laboratories, Westbrook, ME, USA). Proteinuric cats were treated with telmisartan at a dosage of 1 mg/kg/day.^41^ In cats with persistent hyperphosphatemia (serum phosphorus concentration > 4.6 mg/dL) despite dietary restriction, lanthanum carbonate (Antax® 500 mg, Vilsan Pharmaceuticals, Ankara, Turkey) was administered PO at a dosage of 12.5 mg/kg/day, mixed with food.^43^ Lanthanum treatment had been initiated at least 4 weeks before study enrollment and was continued unchanged throughout the 60-day study. For hypercalcemia (serum total calcium concentration > 12 mg/dL), the renal diet was mixed 1:1 with a maintenance diet to decrease calcium intake.^44^ Hypokalemic cats received potassium gluconate or potassium citrate at a dosage of 1-2 mmol/kg/day PO.^24^ In cases of anorexia or weight loss, mirtazapine (Zestat® 15 mg, Sanofi Aventis, Istanbul, Turkey) was administered at a dosage of 1.88 mg/cat every 48 h.^45^ For vomiting, maropitant citrate (Cerenia®, Zoetis Inc., Kalamazoo, MI, USA) was administered at a dosage of 1 mg/kg.^46^ Cats diagnosed with anemia (hematocrit < 20%) on day 0 received darbepoetin alfa (Aranesp®, Amgen İlaç Tic. Ltd. Şti., Istanbul, Turkey) at a dosage of 0.5 μg/kg SC once weekly. All cats treated with darbepoetin also received iron supplementation (ferrous sulfate, 10 mg/kg PO q48h) to prevent iron deficiency and optimize erythropoietic response. The need for ongoing treatment was evaluated on days 15, 30, 45, and 60.^47^

Cats in the MT + C Group (*n* = 8) received the same medical treatment described above, along with an PO curcumin supplementation (Solgar® Full Spectrum Curcumin, Istanbul, Turkey) at a dosage of 80 mg/cat/day (2 divided doses) for 60 consecutive days.^40^ This formulation designed for humans contains micronized curcumin in liquid form (40 mg curcumin per capsule), which provides enhanced gastrointestinal absorption compared with conventional powder forms. The supplement was administered with food to ensure co-ingestion with dietary fat, thereby improving bioavailability. Cats in the Control Group (*n* = 8) did not receive any medical treatment.

#### Sample collection

Blood and urine samples were collected from all cats for eligibility assessment and longitudinal follow-up. In the control group, single blood and urine samples were obtained only at baseline (day 0) to provide physiologic results for contextual comparison. In the MT and MT + C groups, samples were collected at 5 time points: day 0 (before treatment) and on days 15, 30, 45, and 60. Approximately 5 mL of blood was drawn from the jugular vein at each time point and distributed as follows: 0.5 mL into EDTA tubes for hematologic analysis, 0.5 mL into lithium-heparin tubes for plasma-based assays, and 4 mL into serum tubes for biochemical and molecular analyses. Serum was separated by centrifugation at 1006 × g (equivalent to 3000 rpm) for 10 minutes and stored at −80 °C until testing (Esco Lexicon® ULT Freezer, Korea). Urine samples were obtained at the same time points (days 0, 15, 30, 45, and 60) by ultrasound-guided cystocentesis using a 22G needle and 10 mL syringe.

#### Routine laboratory analyses

Hematologic variables including WBC, red blood cell count (RBC), hemoglobin concentration, and hematocrit were measured from EDTA-anticoagulated blood samples using a veterinary hematology analyzer (Mindray BC-500, Mindray Bio-Medical Electronics Co., Shenzhen, China). Serum biochemical variables such as creatinine, phosphorus (P), calcium (Ca), sodium (Na), potassium (K), and total carbon dioxide (TCO₂) were analyzed using a fully automated biochemistry analyzer (Fuji NX 500 I, Fujifilm Corporation, Tokyo, Japan). Serum symmetric dimethylarginine (SDMA) and urine protein-to-creatinine ratio (UPC) were measured using the IDEXX Catalyst One chemistry analyzer (IDEXX Laboratories, Westbrook, ME, USA). Urine specific gravity was assessed using a veterinary refractometer, which was calibrated with distilled water before each use. Azotemic cats with USG > 1.035 were classified as having prerenal azotemia.

#### Oxidative stress analyses

Serum samples were analyzed to assess oxidative status using commercially available colorimetric assay kits for TAS and TOS (Rel Assay Diagnostics, Gaziantep, Turkey; REF: RL0017 for TAS, REF: RL0024 for TOS). All measurements were performed in the Department of Biochemistry, Faculty of Veterinary Medicine, Atatürk University, using an ELISA microplate reader (BioTek Instruments, Winooski, VT, USA). The TAS assay was calibrated using Trolox, and results were expressed in mmol Trolox equivalents per liter (mmol Trolox equiv/L), as previously described.^48^ The TOS assay was using hydrogen peroxide, and results were expressed in mmol hydrogen peroxide equivalents per liter (mmol H₂O₂ equiv/L), as described previously.^49^ All analyses were conducted in duplicate according to the manufacturer’s instructions.

#### Inflammatory and apoptotic marker analyses

Serum concentrations of NF-κB and caspase-3 were measured using feline-specific ELISA kits (Cat NF-κB ELISA Kit, REF: DZE201280411; Cat Caspase-3 ELISA Kit, REF: DZE201281041; Sunred Biological Technology Co., Ltd., Shanghai, China). The assays were conducted at the Department of Biochemistry, Faculty of Veterinary Medicine, Atatürk University, using a microplate reader (BioTek Instruments, Winooski, VT, USA). All procedures were performed in strict accordance with the manufacturer’s instructions, and each sample was analyzed in duplicate to ensure accuracy and reproducibility.

All ELISA kits used in the study (TAS, TOS, NF-κB, and caspase-3) were manufactured under good laboratory practice standards and validated for veterinary or research use by their respective producers. Therefore, no additional in-house validation was required beyond standard calibration and duplicate verification. Detailed analytical characteristics, including sensitivity, assay range, and intra and inter-assay coefficients of variation, are provided in Table S1.

#### Statistical analysis

All statistical analyses were performed using IBM SPSS Statistics version 25.0 (IBM Corp., Armonk, NY, USA). Data initially were assessed for normality using the Shapiro–Wilk test. Parametric data were expressed as mean ± SD, whereas non-parametric data were expressed as median (minimum-maximum). Baseline comparisons among the 3 groups (Control, MT, and MT + C) at day 0 were performed using one-way analysis of variance (ANOVA) for normally distributed variables, followed by Tukey’s post hoc test. Non-normally distributed variables were analyzed using the Kruskal–Wallis test, and pairwise comparisons were performed using the Mann–Whitney U test with Bonferroni correction. Healthy controls were sampled only at baseline (day 0). Therefore, all comparisons involving the healthy control group were restricted to baseline analyses aimed at characterizing disease-associated differences at study entry. Longitudinal treatment effects were evaluated only in the 2 CKD groups (MT and MT + C). For these repeated measurements, longitudinal outcomes were analyzed using linear mixed effects models to account for within-cat correlation and missing observations caused by loss to follow-up. Cat identity was included as a random intercept. Fixed effects included treatment group (MT vs MT + C), time (day 0, 15, 30, 45, 60), and the group × time interaction. Model assumptions were assessed by inspection of residual plots; if needed, outcomes were log-transformed to improve normality and homoscedasticity.

Multiplicity adjustment was applied separately for baseline cross-sectional comparisons and for longitudinal post hoc comparisons. The primary endpoint was SDMA. Secondary prespecified endpoints were UPC, TAS, TOS, NF-κB, and caspase-3; all additional laboratory variables and SBP were treated as exploratory. Baseline comparisons involving the healthy control group were considered supportive analyses for disease characterization, whereas efficacy analyses were based on longitudinal comparisons between the MT and MT + C groups. For each outcome, post hoc pairwise comparisons across time points or between groups at specific time points in the longitudinal analyses were adjusted using Bonferroni correction within that outcome. Two-sided *P*-values < .05 were considered statistically significant for the primary endpoint; secondary and exploratory analyses were interpreted as hypothesis-generating.

## Off-label antimicrobial declaration

Authors declare no off-label use of antimicrobials.

## Institutional animal care and use committee or other approval declaration

Approved by the Atatürk University Animal Experiments Local Ethics Committee, approval number: 66, approval date: April 12, 2021, meeting number: 3.

## Abbreviations

ANOVA: analysis of variance
Bcl-2: B-cell lymphoma 2
CAT: catalase
CKD: chronic kidney disease
FGF-23: fibroblast growth factor 23
GFR: glomerular filtration rate
GPx: glutathione peroxidase
IκBα: inhibitor of kappa B alpha
IL-1β: interleukin-1 beta
IL-6: interleukin-6
MT: medical treatment
MT + C: medical treatment plus curcumin
NO: nitric oxide
NF-κB: nuclear factor kappa B
ROS: reactive oxygen species
SDMA: serum symmetric dimethylarginine
SOD: superoxide dismutase
SBP: systolic blood pressure
TAS: total antioxidant status
TCO₂: total carbon dioxide
TOS: total oxidant status
TNF-α: tumor necrosis factor alpha
UPC: urine protein-to-creatinine ratio
USG: urine specific gravity

## References

[1] Polzin DJ . Chronic kidney disease in small animals. Vet Clin North Am Small Anim Pract. 2011;41:15-30. 10.1016/j.cvsm.2010.09.004 21251509

[2] Chakrabarti S, Syme HM, Brown CA, Elliott J. Histomorphometry of feline chronic kidney disease and correlation with markers of renal dysfunction. Vet Pathol. 2013;50:147-155. 10.1177/0300985812453176 22773469

[3] International Renal Interest Society 2016. https://www.iris-kidney.com/iris-guidelines-1.

[4] King JN, Tasker S, Gunn-Moore DA, Strehlau G. Benrıc (benazepril in renal insufficiency in cats) study group. Prognostic factors in cats with chronic kidney disease. J Vet Intern Med. 2007;21:906-916. 10.1111/j.1939-1676.2007.tb03042.x 17939542

[5] Chakrabarti S, Syme HM, Elliott J. Clinicopathological variables predicting progression of azotemia in cats with chronic kidney disease. J Vet Intern Med. 2012;26:275-281. 10.1111/j.1939-1676.2011.00874.x 22269057

[6] Hall JA, Yerramilli M, Obare E, Yerramilli M, Jewell DE. Comparison of serum concentrations of symmetric dimethylarginine and creatinine as kidney function biomarkers in cats with chronic kidney disease. J Vet Intern Med. 2014;28:1676-1683. 10.1111/jvim.12445 25231385 PMC4895610

[7] Braff J, Obare E, Yerramilli M, Elliott J, Yerramilli M. Relationship between serum symmetric dimethylarginine concentration and glomerular filtration rate in cats. J Vet Intern Med. 2014;28:1699-1701. 10.1111/jvim.12446 25272985 PMC4895615

[8] Jepson RE, Elliott J, Brodbelt D, Syme HM. Effect of control of systolic blood pressure on survival in cats with systemic hypertension. J Vet Intern Med. 2007;21:402-409. 10.1111/j.1939-1676.2007.tb02982.x 17552443

[9] Syme HM, Markwell PJ, Pfeiffer D, Elliott J. Survival of cats with naturally occurring chronic renal failure is related to severity of proteinuria. J Vet Intern Med. 2006;20:528-535. 10.1111/j.1939-1676.2006.tb02892.x 16734085

[10] Boyd LM, Langston C, Thompson K, Zivin K, Imanishi M. Survival in cats with naturally occurring chronic kidney disease (2000-2002). J Vet Intern Med. 2008;22:1111-1117. 10.1111/j.1939-1676.2008.0163.x 18691369

[11] Daenen K, Andries A, Mekahli D, Van Schepdael A, Jouret F, Bammens B. Oxidative stress in chronic kidney disease. Pediatr Nephrol. 2019;34:975-991. 10.1007/s00467-018-4005-4 30105414

[12] Ruiz S, Pergola PE, Zager RA, Vaziri ND. Targeting the transcription factor Nrf2 to ameliorate oxidative stress and inflammation in chronic kidney disease. Kidney Int. 2013;83:1029-1041. 10.1038/ki.2012.439 23325084 PMC3633725

[13] Rapa SF, Di Iorio BR, Campiglia P, Heidland A, Marzocco S. Inflammation and oxidative stress in chronic kidney disease-potential therapeutic role of minerals, vitamins and plant-derived metabolites. Int J Mol Sci. 2019;21:263. 10.3390/ijms21010263 31906008 PMC6981831

[14] Tapia E, Soto V, Ortiz-Vega KM, et al. Curcumin induces Nrf2 nuclear translocation and prevents glomerular hypertension, hyperfiltration, oxidant stress, and the decrease in antioxidant enzymes in 5/6 nephrectomized rats. Oxid Med Cell Longev. 2012;2012:1-14. 10.1155/2012/269039 PMC3424005 22919438

[15] Gupta SC, Patchva S, Koh W, Aggarwal BB. Discovery of curcumin, a component of golden spice, and its miraculous biological activities. Clin Exp Pharmacol Physiol. 2012;39:283-299. 10.1111/j.1440-1681.2011.05648.x 22118895 PMC3288651

[16] Zhao Y-H, Shen C-F, Kang Y, et al. Curcumin prevents renal cell apoptosis in acute kidney injury in a rat model of dry-heat environment heatstroke via inhibition of the mitochondrial apoptotic pathway. Exp Ther Med. 2021;21:126. 10.3892/etm.2020.9558 33376508 PMC7751465

[17] Soetikno V, Sari FR, Veeraveedu PT, et al. Curcumin ameliorates macrophage infiltration by inhibiting NF-κB activation and proinflammatory cytokines in streptozotocin induced-diabetic nephropathy. Nutr Metab (Lond). 2011;8:35. 10.1186/1743-7075-8-35 21663638 PMC3123175

[18] Menon VP, Sudheer AR. Antıoxıdant and anti-inflammatory properties of curcumin. In: Bharat BA, Young JS, Shishir S, eds. The Molecular Targets and Therapeutic Uses of Curcumin in Health and Disease. Springer US; n.d:105-125. 10.1007/978-0-387-46401-5_3.

[19] Shoba G, Joy D, Joseph T, Majeed M, Rajendran R, Srinivas P. Influence of piperine on the pharmacokinetics of curcumin in animals and human volunteers. Planta Med. 1998;64:353-356. 10.1055/s-2006-957450 9619120

[20] Anand P, Kunnumakkara AB, Newman RA, Aggarwal BB. Bioavailability of curcumin: problems and promises. Mol Pharm. 2007;4:807-818. 10.1021/mp700113r 17999464

[21] Hokamp JA, Nabity MB. Renal biomarkers in domestic species. Vet Clin Pathol. 2016;45:28-56. 10.1111/vcp.12333 26918420

[22] Zhang J, Tang L, Sen LG, Wang J. The anti-inflammatory effects of curcumin on renal ischemia-reperfusion injury in rats. Ren Fail. 2018;40:680-686. 10.1080/0886022X.2018.1544565 30741618 PMC6282432

[23] Soetikno V, Sari FR, Lakshmanan AP, et al. Curcumin alleviates oxidative stress, inflammation, and renal fibrosis in remnant kidney through the Nrf2-keap1 pathway. Mol Nutr Food Res. 2013;57:1649-1659. 10.1002/mnfr.201200540 23174956

[24] Bartges JW . Chronic kidney disease in dogs and cats. Vet Clin North Am Small Anim Pract. 2012;42:669-692. 10.1016/j.cvsm.2012.04.008 22720808

[25] Sparkes AH, Caney S, Chalhoub S, et al. ISFM consensus guidelines on the diagnosis and management of feline chronic kidney disease. J Feline Med Surg. 2016;18:219-239. 10.1177/1098612X16631234 26936494 PMC11148907

[26] Geddes RF, Elliott J, Syme HM. The effect of feeding a renal diet on plasma fibroblast growth factor 23 concentrations in cats with stable azotemic chronic kidney disease. J Vet Intern Med. 2013;27:1354-1361. 10.1111/jvim.12187 24010686

[27] Geddes RF, Finch NC, Syme HM, Elliott J. The role of phosphorus in the pathophysiology of chronic kidney disease. J Vet Emerg Crit Care. 2013;23:122-133. 10.1111/vec.12032 23464730

[28] Ali BH, Al-Salam S, Al Suleimani Y, et al. Curcumin ameliorates kidney function and oxidative stress in experimental chronic kidney disease. Basic Clin Pharmacol Toxicol. 2018;122:65-73. 10.1111/bcpt.12817 28561324

[29] Grauer GF . Proteinuria: measurement and interpretation. Top Companion Anim Med. 2011;26:121-127. 10.1053/j.tcam.2011.04.002 21782142

[30] Lees GE, Brown SA, Elliott J, Grauer GF, Vaden SL, American College of Veterinary Internal Medicine. Assessment and management of proteinuria in dogs and cats: 2004 ACVIM forum consensus statement (small animal). J Vet Intern Med. 2005;19:377-385. 10.1111/j.1939-1676.2005.tb02713.x 15954557

[31] Ghosh S, Gehr T, Ghosh S. Curcumin and chronic kidney disease (CKD): major mode of action through stimulating endogenous intestinal alkaline phosphatase. Molecules. 2014;19:20139-20156. 10.3390/molecules191220139 25474287 PMC6271001

[32] Aggarwal BB, Sundaram C, Malani N, Ichikawa H. Curcumın: The Indıan Solıd Gold. In: Bharat BA, Chitra S, Nikita M, Haruya I, eds. The Molecular Targets and Therapeutic Uses of Curcumin in Health and Disease*.* Springer US; n.d:1-75. 10.1007/978-0-387-46401-5_1

[33] Kalpana C, Menon VP. Curcumin ameliorates oxidative stress during nicotine-induced lung toxicity in Wistar rats. Ital J Biochem. 2004;53:82-86. 15646012

[34] Jobin C, Bradham CA, Russo MP, et al. Curcumin blocks cytokine-mediated NF-kappa B activation and proinflammatory gene expression by inhibiting inhibitory factor I-kappa B kinase activity. J Immunol. 1999;163:3474-3483. 10.4049/jimmunol.163.6.3474 10477620

[35] Pila P, Chuammitri P, Patchanee P, Pringproa K, Piyarungsri K. Evaluation of Bcl-2 as a marker for chronic kidney disease prediction in cats. Front Vet Sci. 2023;9:9. 10.3389/fvets.2022.1043848 PMC9870326 36699321

[36] Brown CA, Elliott J, Schmiedt CW, Brown SA. Chronic kidney disease in aged cats. Vet Pathol. 2016;53:309-326. 10.1177/0300985815622975 26869151

[37] Jiang A-J, Jiang G, Li L-T, Zheng J-N. Curcumin induces apoptosis through mitochondrial pathway and caspases activation in human melanoma cells. Mol Biol Rep. 2015;42:267-275. 10.1007/s11033-014-3769-2 25262359

[38] Xu X-Y, Meng X, Li S, Gan R-Y, Li Y, Li HB. Bioactivity, health benefits, and related molecular mechanisms of curcumin: current progress, challenges, and perspectives. Nutrients. 2018;10:2–33. 10.3390/nu10101553 PMC6213156 30347782

[39] Zhao L, Gu Q, Xiang L, et al. Curcumin inhibits apoptosis by modulating Bax/Bcl-2 expression and alleviates oxidative stress in testes of streptozotocin-induced diabetic rats. Ther Clin Risk Manag. 2017;13:1099-1105. 10.2147/TCRM.S141738 28894373 PMC5584885

[40] Fougere B . Popular Thai culinary herbs and how they can help companion animals. In: 40th World Small Anim. Vet. Assoc. Congr. Bangkok, Thailand, 15-18 May, 2015. Proc. B. 2015:274-275.

[41] Lees GE, Brown SA, Elliott J, Grauer GE, Vaden SL, American College of Veterinary Internal Medicine. Assessment and management of proteinuria in dogs and cats: 2004 ACVIM forum consensus statement (small animal). J Vet Intern Med. 2005;19:377-385. 10.1892/0891-6640(2005)19[377:aamopi]2.0.co;2 15954557

[42] Acierno MJ, Brown S, Coleman AE, et al. ACVIM consensus statement: guidelines for the identification, evaluation, and management of systemic hypertension in dogs and cats. J Vet Intern Med. 2018;32:1803-1822. 10.1111/jvim.15331 30353952 PMC6271319

[43] Chew DJ . Chronic kidney disease (CKD) in dogs & cats-staging and management strategies. Va Vet Med Assoc. 2015;1:1-22

[44] van den Broek DHN, Chang Y-M, Elliott J, Jepson RE. Chronic kidney disease in cats and the risk of total hypercalcemia. J Vet Intern Med. 2017;31:465-475. 10.1111/jvim.14643 28190275 PMC5354036

[45] Quimby JM, Lunn KF. Mirtazapine as an appetite stimulant and anti-emetic in cats with chronic kidney disease: a masked placebo-controlled crossover clinical trial. Vet J. 2013;197:651-655. 10.1016/j.tvjl.2013.05.048 23838205

[46] Martin-Flores M, Sakai DM, Mastrocco A, et al. Evaluation of oral maropitant as an antiemetic in cats receiving morphine and dexmedetomidine. J Feline Med Surg. 2016;18:921-924. 10.1177/1098612X15613389 26534944 PMC11132224

[47] Chalhoub S, Langston CE, Farrelly J. The use of darbepoetin to stimulate erythropoiesis in anemia of chronic kidney disease in cats: 25 cases. J Vet Intern Med. 2012;26:363-369. 10.1111/j.1939-1676.2011.00864.x 22296687

[48] Erel O . A novel automated method to measure total antioxidant response against potent free radical reactions. Clin Biochem. 2004;37:112-119. 10.1016/j.clinbiochem.2003.10.014 14725941

[49] Erel O . A new automated colorimetric method for measuring total oxidant status. Clin Biochem. 2005;38:1103-1111. 10.1016/j.clinbiochem.2005.08.008 16214125

