# Supplementary material for: Renoprotective effects of curcumin in cats with chronic kidney disease
Source: J Vet Intern Med. 2026 Jul 16;40(4):aalag138. doi: 10.1093/jvimsj/aalag138 (PMC13375107; doi:10.1093/jvimsj/aalag138)
Supplement: R1_Supplementary_Table_S1_aalag138 [file r1_supplementary_table_s1_aalag138.docx]

| **Assay** | **Manufacturer** | **REF** | **GLP Compliance** | **Detection Limit** | **Intra-assay CV (%)** | **Inter-assay CV (%)** | **Assay Principle** |
| --- | --- | --- | --- | --- | --- | --- | --- |
| **Total Antioxidant Status (TAS)** | Rel Assay Diagnostics, Turkey | RL0017 | Yes | 0.01 – 4.00 mmol Trolox equiv/L | 3.3% | 2.8% | Colorimetric |
| **Total Oxidant Status (TOS)** | Rel Assay Diagnostics, Turkey | RL0024 | Yes | 0.02 – 80 µmol H₂O₂ equiv/L | 3.9% | 3.2% | Colorimetric |
| **Cat NF-κB ELISA** | Sunred Biological Technology, China | DZE201280411 | Yes | 0.08–20 ng/mL | <10% | <12% | Sandwich ELISA |
| **Cat Caspase-3 ELISA** | Sunred Biological Technology, China | DZE201281041 | Yes | 0.06–18 ng/mL | <10% | <10% | Sandwich ELISA |

#### **Supplementary Table 1** Analytical performance characteristics of the assays used in this study
