# Supplementary material for: Renoprotective effects of curcumin in cats with chronic kidney disease
Source: J Vet Intern Med. 2026 Jul 16;40(4):aalag138. doi: 10.1093/jvimsj/aalag138 (PMC13375107; doi:10.1093/jvimsj/aalag138)
Supplement: Supplementary_Methods_S1_aalag138 [file supplementary_methods_s1_aalag138.docx]

**2. Materials and methods**

***2.1 Ethical Approval***

Our study was conducted in accordance with the ethical principles for animal experimentation and was approved by the Atatürk University Animal Experiments Local Ethics Committee (approval no. 66; approval date: 12.04.2021; meeting no. 3). Written informed consent was obtained from the owners of all participating cats before inclusion in the study.

***2.2 Animals***

Twenty-four client-owned domestic cats were enrolled in the study, including 8 clinically healthy controls and 16 cats with IRIS stage 3 CKD. Chronic kidney disease diagnosis was based on persistent azotemia and clinical findings, and IRIS staging was performed exclusively according to serum creatinine concentrations (2.9-5.0 mg/dL for Stage 3). Concentrations of SDMA (≥26 μg/dL) were evaluated as supportive biomarkers to confirm chronic renal dysfunction but were not used for staging. Cats with prerenal azotemia were stabilized before assessment, and findings inconsistent with CKD (e.g., normal SDMA with persistent isosthenuria) were excluded. Diagnostic criteria also included persistent isosthenuria (urine specific gravity <1.035) and clinical signs such as polyuria, polydipsia, weight loss, and decreased appetite. All CKD cats were clinically stable and not dehydrated at the time of staging, and IRIS classification was performed after stabilization of renal variables. Cats presented with prerenal azotemia or dehydration were initially treated using fluid therapy and re-evaluated after 72 hours before inclusion. Most CKD cases were previously diagnosed within the previous 3 months and had been maintained on a commercial renal diet before study enrollment. Cats with concurrent systemic diseases (e.g., hyperthyroidism, diabetes mellitus, neoplasia) or recent use of antioxidant or anti-inflammatory supplements were excluded. Before enrollment, all animals underwent comprehensive physical examination, CBC, serum biochemistry, urinalysis, and abdominal ultrasonography.

***2.3 Study Design and Group Allocation***

The primary outcome of the study was the longitudinal change in serum SDMA concentrations between the MT and MT+C groups, selected because of its clinical relevance as a sensitive marker of renal function in cats with CKD. Secondary outcomes included UPC, oxidative stress markers (TAS, TOS), and inflammatory and apoptotic biomarkers (NF-κB, Caspase-3), which were assessed to provide mechanistic context for the primary endpoint.

Twenty-four cats were assigned into three equal groups (n = 8 per group) as follows:

Control Group (Control): Clinically healthy cats without evidence of CKD, as confirmed by normal physical examination findings, normal hematologic and biochemical test results, urine specific gravity >1.035, and normal renal ultrasonographic appearance. The control group was included to facilitate baseline physiological comparisons with the CKD groups and to contextualize disease-associated alterations in biomarker profiles.

Medical treatment group (MT): Cats diagnosed with IRIS stage 3 CKD received medical treatment only, including a renal prescription diet, fluid therapy, phosphate binders, and antihypertensive agents, without any additional supplementation.

Curcumin Group (MT + C): Cats with stage 3 CKD received the same medical treatment as the MT group, and PO curcumin supplementation (Solgar® Full Spectrum Curcumin, Istanbul, Turkey) at a dosage of 80 mg/cat/day (two divided doses) for 60 consecutive days [21]. This formulation, designed for humans, contained micronized curcumin in liquid form (40 mg curcumin per capsule) and was administered with food to enhance absorption.

Cats were treated and monitored at a Pethome Veterinary Polyclinic. Randomization was conducted using a computer-generated sequence. No placebo was administered to the MT group but all investigators and laboratory personnel were blinded to group allocation throughout the study.

The healthy control group was included to permit baseline cross-sectional comparisons with the CKD groups and to characterize disease-related alterations in oxidative, inflammatory, and apoptotic biomarkers at study entry, especially because standardized reference ranges for several of these biomarkers are not well established in cats. However, the healthy control group was not intended to serve as a comparator for treatment efficacy. Therefore, evaluation of therapeutic effects was based primarily on longitudinal comparisons between the MT and MT + C groups.

***2.4 Treatment Protocols***

Throughout the study, cats in the medical treatment group (MT, n = 8) diagnosed with stage 3 CKD received comprehensive medical management in accordance with IRIS guidelines. All treatments were performed at a Pethome Veterinary Polyclinic. All cats were fed a renal prescription diet (Unique Renal® Diet, France) beginning on day 0. In dehydrated cats, Ringer’s lactate solution was administered IV based on body weight, followed by SC fluid administration every other day for maintenance. Metabolic acidosis was determined by measuring total carbon dioxide (TCO_2_) concentrations using an automated chemistry analyzer (Fuji NX 500 I, Fujifilm Corporation, Tokyo, Japan), and cats with TCO₂ concentrations <15 mmol/L were considered acidotic [1]. Systolic blood pressure (SBP) was measured on days 0, 15, 30, 45, and 60 using a high-definition oscillometric device (Hasvet 838PM, Hasvet Medical, Istanbul, Turkey). Measurements were obtained with the cat in sternal recumbency, using an appropriately sized cuff (40% of limb circumference) placed on the median portion of the forelimb. All measurements were performed in a quiet room after a 5-minute acclimation period to minimize stress-induced variability. For each time point, five consecutive readings were recorded, and the mean value of the last three measurements was used for analysis. Cats with systolic blood pressure ≥160 mmHg received telmisartan (Semintra® 4 mg/mL, Boehringer Ingelheim Vetmedica, Ingelheim, Germany) at a dosage of 2 mg/kg/day to decrease systolic pressure below 160 mmHg [22,23]. Serum creatinine concentration and UPC ratios were evaluated on days 0, 15, 30, 45, and 60. Creatinine concentrations were measured using a biochemistry analyzer (Fuji NX 500 I, Fujifilm Corporation, Tokyo, Japan) and UPC ratios were determined using an automated chemistry analyzer (Catalyst One, IDEXX Laboratories, Westbrook, ME, USA). Proteinuric cats were treated with telmisartan at a dosage of 1 mg/kg/day [22]. In cats with persistent hyperphosphatemia (serum phosphorus concentration >4.6 mg/dL) despite dietary restriction, lanthanum carbonate (Antax® 500 mg, Vilsan Pharmaceuticals, Ankara, Turkey) was administered PO at a dosage of 12.5 mg/kg/day, mixed with food [24]. Lanthanum treatment had been initiated at least four weeks before study enrollment and was continued unchanged throughout the 60-day study. For hypercalcemia (serum total calcium concentration >12 mg/dL), the renal diet was mixed 1:1 with a maintenance diet to decrease calcium intake [25]. Hypokalemic cats received potassium gluconate or potassium citrate at a dosage of 1-2 mmol/kg/day PO [26]. In cases of anorexia or weight loss, mirtazapine (Zestat® 15 mg, Sanofi Aventis, Istanbul, Turkey) was administered at a dosage of 1.88 mg/cat every 48 hours [27]. For vomiting, maropitant citrate (Cerenia®, Zoetis Inc., Kalamazoo, MI, USA) was administered at a dosage of 1 mg/kg [28]. Cats diagnosed with anemia (hematocrit <20%) on day 0 received darbepoetin alfa (Aranesp®, Amgen İlaç Tic. Ltd. Şti., Istanbul, Turkey) at a dosage of 0.5 µg/kg SC once weekly. All cats treated with darbepoetin also received iron supplementation (ferrous sulfate, 10 mg/kg PO q48h) to prevent iron deficiency and optimize erythropoietic response. The need for ongoing treatment was evaluated on days 15, 30, 45, and 60 [29].

Cats in the MT + C Group (n = 8) received the same medical treatment described above, along with an PO curcumin supplementation (Solgar® Full Spectrum Curcumin, Istanbul, Turkey) at a dosage of 80 mg/cat/day (two divided doses) for 60 consecutive days [21]. This formulation designed for humans contains micronized curcumin in liquid form (40 mg curcumin per capsule), which provides enhanced gastrointestinal absorption compared with conventional powder forms. The supplement was administered with food to ensure co-ingestion with dietary fat, thereby improving bioavailability. Cats in the Control Group (n = 8) did not receive any medical treatment.

***2.5 Sample Collection***

Blood and urine samples were collected from all cats for eligibility assessment and longitudinal follow-up. In the control group, single blood and urine samples were obtained only at baseline (day 0) to provide physiologic results for contextual comparison. In the MT and MT + C groups, samples were collected at five time points: day 0 (before treatment) and on days 15, 30, 45, and 60. Approximately 5 mL of blood was drawn from the jugular vein at each time point and distributed as follows: 0.5 mL into EDTA tubes for hematologic analysis, 0.5 mL into lithium-heparin tubes for plasma-based assays, and 4 mL into serum tubes for biochemical and molecular analyses. Serum was separated by centrifugation at 3000 rpm for 10 minutes and stored at -80°C until testing (Esco Lexicon® ULT Freezer, Korea). Urine samples were obtained at the same time points (days 0, 15, 30, 45, and 60) by ultrasound-guided cystocentesis using a 22G needle and 10 mL syringe.

***2.6 Routine Laboratory Analyses***

Hematologic variables including white blood cell count (WBC), red blood cell count (RBC), hemoglobin concentration (HGB), and hematocrit (HCT) were measured from EDTA-anticoagulated blood samples using a veterinary hematology analyzer (Mindray BC-500, Mindray Bio-Medical Electronics Co., Shenzhen, China). Serum biochemical variables such as creatinine, phosphorus (P), calcium (Ca), sodium (Na), potassium (K), and total carbon dioxide (TCO₂) were analyzed using a fully automated biochemistry analyzer (Fuji NX 500 I, Fujifilm Corporation, Tokyo, Japan). Serum symmetric dimethylarginine (SDMA) and urine protein-to-creatinine ratio (UPC) were measured using the IDEXX Catalyst One chemistry analyzer (IDEXX Laboratories, Westbrook, ME, USA). Urine specific gravity (USG) was assessed using a veterinary refractometer, which was calibrated with distilled water before each use. Azotemic cats with USG > 1.035 were classified as having prerenal azotemia.

***2.7 Oxidative Stress Analyses***

Serum samples were analyzed to assess oxidative status using commercially available colorimetric assay kits for total antioxidant status (TAS) and total oxidant status (TOS); (Rel Assay Diagnostics, Gaziantep, Turkey; REF: RL0017 for TAS, REF: RL0024 for TOS). All measurements were performed in the Department of Biochemistry, Faculty of Veterinary Medicine, Atatürk University, using an ELISA microplate reader (BioTek Instruments, Winooski, VT, USA). The TAS assay was calibrated using Trolox, and results were expressed in mmol Trolox equivalents per liter (mmol Trolox equiv/L), as previously described [30]. The TOS assay was using with hydrogen peroxide, and results were expressed in mmol hydrogen peroxide equivalents per liter (mmol H₂O₂ equiv/L), as described previously [31]. All analyses were conducted in duplicate according to the manufacturer’s instructions.

***2.8 Inflammatory and Apoptotic Marker Analyses***

Serum concentrations of NF-κB and caspase-3 were measured using feline-specific ELISA kits (Cat NF-κB ELISA Kit, REF: DZE201280411; Cat Caspase-3 ELISA Kit, REF: DZE201281041; Sunred Biological Technology Co., Ltd., Shanghai, China). The assays were conducted at the Department of Biochemistry, Faculty of Veterinary Medicine, Atatürk University, using a microplate reader (BioTek Instruments, Winooski, VT, USA). All procedures were performed in strict accordance with the manufacturer’s instructions, and each sample was analyzed in duplicate to ensure accuracy and reproducibility.

All ELISA kits used in the study (TAS, TOS, NF-κB, and caspase-3) were manufactured under good laboratory practice (GLP) standards and validated for veterinary or research use by their respective producers. Therefore, no additional in-house validation was required beyond standard calibration and duplicate verification. Detailed analytical characteristics, including sensitivity, assay range, and intra and inter-assay coefficients of variation, are provided in Supplementary Table S1.

***2.9 Statistical Analysis***

All statistical analyses were performed using IBM SPSS Statistics version 25.0 (IBM Corp., Armonk, NY, USA). Data assessed for normality using the Shapiro-Wilk test. Parametric data were expressed as mean ± SD, whereas non-parametric data were expressed as median (minimum-maximum). Baseline comparisons among the three groups (Control, MT, and MT + C) at day 0 were performed using one-way analysis of variance (ANOVA) for normally distributed variables, followed by Tukey’s post hoc test. Non-normally distributed variables were analyzed using the Kruskal-Wallis test, and pairwise comparisons were performed using the Mann-Whitney U test with Bonferroni correction. Healthy controls were sampled only at baseline (day 0). Therefore, all comparisons involving the healthy control group were restricted to baseline analyses aimed at characterizing disease-associated differences at study entry. Longitudinal treatment effects were evaluated only in the two CKD groups (MT and MT + C). For these repeated measurements, longitudinal outcomes were analyzed using linear mixed effects models to account for within-cat correlation and missing observations caused by loss to follow-up. Cat identity was included as a random intercept. Fixed effects included treatment group (MT vs MT + C), time (day 0, 15, 30, 45, 60), and the group × time interaction. Model assumptions were assessed by inspection of residual plots; if needed, outcomes were log-transformed to improve normality and homoscedasticity.

Multiplicity adjustment was applied separately for baseline cross-sectional comparisons and for longitudinal post hoc comparisons. The primary endpoint was SDMA. Secondary prespecified endpoints were UPC, TAS, TOS, NF-κB, and caspase-3; all additional laboratory variables and systolic blood pressure were treated as exploratory. Baseline comparisons involving the healthy control group were considered supportive analyses for disease characterization, whereas efficacy analyses were based on longitudinal comparisons between the MT and MT + C groups. For each outcome, post hoc pairwise comparisons across time points or between groups at specific time points in the longitudinal analyses were adjusted using Bonferroni correction within that outcome. Two-sided P values < .05 were considered statistically significant for the primary endpoint; secondary and exploratory analyses were interpreted as hypothesis-generating.
